# Incidence and Genetic Diversity of Grapevine Virus G in Croatian Vineyards

**DOI:** 10.3390/plants11182341

**Published:** 2022-09-07

**Authors:** Martin Jagunić, Alfredo Diaz-Lara, Lóránt Szőke, Maher Al Rwahnih, Kristian Stevens, Goran Zdunić, Darko Vončina

**Affiliations:** 1 Department of Plant Pathology, Faculty of Agriculture, University of Zagreb, 10000 Zagreb, Croatia; 2 School of Engineering and Sciences, Tecnologico de Monterrey, Campus Queretaro, Queretaro 76130, Mexico; 3 Faculty of Agricultural and Food Sciences and Environmental Management, Institute of Food Science, University of Debrecen, 138 Böszörményi St., 4032 Debrecen, Hungary; 4 Department of Plant Pathology, Foundation Plant Services, University of California-Davis, Davis, CA 95616, USA; 5 Computer Science and Evolution and Ecology, University of California-Davis, Davis, CA 95616, USA; 6 Institute for Adriatic Crops and Karst Reclamation, 21000 Split, Croatia; 7 Centre of Excellence for Biodiversity and Molecular Plant Breeding, 10000 Zagreb, Croatia

**Keywords:** grapevine, vitivirus, RT-PCR, detection, field survey, sequencing, phylogeny

## Abstract

Grapevine virus G (GVG) is a recently discovered vitivirus infecting grapevines. Historically, viruses in the genus *Vitivirus* have been associated with the grapevine rugose wood disease. Based on new and previously reported GVG isolates, primers and probes were developed for real-time RT-PCR. The developed assay successfully detected the virus in infected plants during dormancy and the growing season. A field study of 4327 grapevines from Croatian continental and coastal wine-growing regions confirmed the presence of GVG in 456 (~10.5%) grapevines from three collection plantations and 77 commercial vineyards, with infection rates ranging from 2% to 100%. Interestingly, the virus was confirmed only in vines considered to be Croatian autochthonous cultivars, but not in introduced cultivars. A 564-nucleotide long portion of the coat protein gene from previously known and newly characterized GVG isolates had nucleotide and amino acid identities ranging from 89% to 100% and from 96.8% to 100%, respectively. Phylogenetic analysis revealed five distinct groups, with isolates originating from the same site being close to each other, indicating possible local infection. The information presented in this manuscript sets the stage for future studies to better understand the ecology and epidemiology of GVG and the possible need for inclusion in certification schemes.

## 1. Introduction

The genus *Vitivirus* (family *Betaflexiviridae*) includes most grapevine-infecting viruses. According to the current taxonomy of the International Committee on Taxonomy of Viruses (ICTV 2021, Master Species List #37), the genus *Vitivirus* consists of 15 viruses, nine of which are grapevine-infecting: grapevine virus A (GVA), grapevine virus B (GVB), grapevine virus D (GVD), grapevine virus E (GVE), grapevine virus F (GVF), grapevine virus G (GVG), grapevine virus H (GVH), grapevine virus I (GVI) and grapevine virus J (GVJ) [1,2,3,4,5,6,7,8,9]. In addition to the previously mentioned viruses, five novel viruses resembling vitiviruses were identified in grapevine by high-throughput sequencing (HTS) and tentatively named grapevine virus K (GVK), grapevine virus L (GVL), grapevine virus M (GVM), grapevine virus N (GVN) and grapevine virus O (GVO) [10,11,12,13]. Vitiviruses are characterized by filamentous particles with single-stranded, positive-sense (+) RNA genomes of approximately 7500 nucleotides, typically containing five open reading frames—ORFs [14].

The best studied ones are also the first grapevine-infecting vitiviruses discovered: GVA and GVB, which were segregated from the genus *Trichovirus* [15]. They are economically important viruses transmitted by contaminated planting material, mechanical inoculation, grafting, and insects (soft scales and mealybugs). Both are causing various changes in grapevine and a disease called rugose wood (RW). Of the several symptoms recognized within the RW complex, GVA is associated with Kober stem grooving (KSG) and GVB with corky bark (CB) [16,17]. Infection of herbaceous test plants with these two viruses has been confirmed, as well as their distribution in all major grape growing regions of the world [18]. On the other hand, GVG was discovered in 2017 by HTS in New Zealand in the grapevine cultivar Chardonnay, originating from France, with a genome length of 7496 nt (GenBank: MF405923) and five ORFs-encoding proteins and containing conserved domains typical for the members of the genus *Vitivirus*. ORF1 encodes a polyprotein, ORF2 encodes a 154 aa long protein, ORF3 comprises the viral movement protein domain, ORF4 encodes the Tricho CP domain of 201 aa, while ORF5 contains the viral nucleic acid binding domain [6]. After the first finding, GVG was detected in Croatia from four autochthonous grapevine cvs. Ljutun, Dobričić, Vlaška and Babica, originating from the Kaštela region [19]. Later, four isolates were found in Pinot noir and Chardonnay in the USA by conventional one-step RT-PCR [20]. Partly because of mixed infection in GVG-infected vines, there are no precise data on the symptomatology induced in contaminated vines.

Although HTS is more sensitive compared to the other detection methods (molecular, serological, or biological) and allows for novel virus detection and screening of a much larger set of samples (pool) in a time and space frame, PCR-based detection methods are still better suited for large-scale viral testing, thus providing much higher detection sensitivity and specificity compared to other diagnostic methods, such as biological indexing and enzyme-linked immunosorbent assays—ELISA [21]. Therefore, the aims of this study were to develop a robust and accurate detection method based on real-time RT-PCR, investigate the possibility of detection during dormancy and the growing season, and determine the genetic structure and distribution of the GVG populations in Croatia. Once revealed, the incidence of GVG in Croatian vineyards, implications and future perspectives were discussed.

## 2. Results

### 2.1. Real-Time RT-PCR Assay, HTS and Bioinformatic Analysis

**Figure 1:**
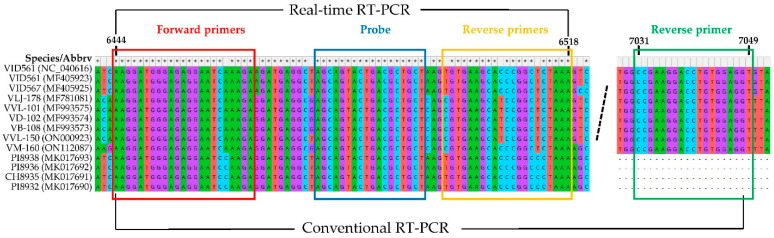
Sequences of different GVG isolates within the portion of the CP region selected for primers and the probe design. The dashed line presents an excised part of the CP region.

After alignment of all 11 available GVG sequences from the GenBank database and the sequences of the two newly characterized GVG isolates via HTS, VVL-150 and VM-160 (Supplementary Table S1), the CP region was selected for primers and probe construction. To enable detection of different GVG isolates, two forward primers designated GVG F1 and GVG F2, three reverse primers designated GVG R1, GVG R2, and GVG R3, and one TaqMan probe designated GVG P were designed for real-time RT-PCR, and their mixture was used for detection, resulting in a product size of 73–75 bps (Figure 1, Table 1).

**Figure 2:**
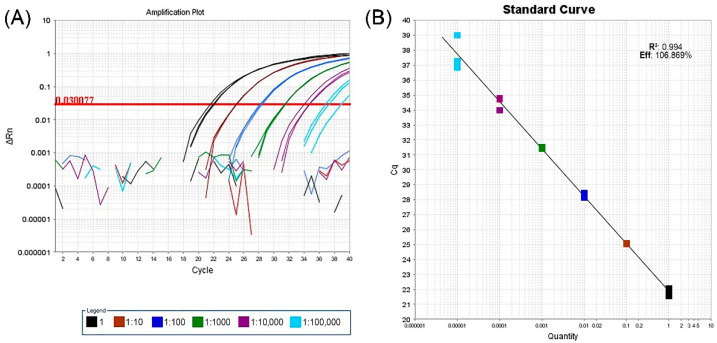
Validation of new real-time RT-PCR assay based on three replicates of ten-fold serial dilutions from a GVG-positive grapevine. 1—undiluted extract; 1:10–1:100,000–10-fold serial dilutions. (**A**) Plot of amplification curves above the set threshold line value. The broken lines below the threshold represent negative controls for each dilution. (**B**) Standard curve of assay sensitivity analysis: x-axis—RNA dilution; y-axis—measured value of Cq; R^2^—coefficient of determination; Eff.- assay efficiency.

A validation test of real-time RT-PCR performed on a GVG-positive grapevine from the Grapevine Virus Collection and processed in three replicates of 10-fold serial dilutions, showed that the assay was able to detect virus down to a dilution of 1:100,000. Analysis of the corresponding standard curves for real-time RT-PCR using the mixture of developed primers and probes indicated that the efficiency was 106.869% with a coefficient determination of 0.994 (Figure 2).

### 2.2. Detection during Dormancy and Growing Season

To determine the potential problematic period for sampling, and consequently to avoid false-negative results during large-scale screening of GVG incidence, detection in different periods of vegetation and dormancy was performed during 2019. As a result, GVG was successfully detected by real-time RT-PCR, with the Cq threshold set at 35, from all five grapevine accessions (VVL 112, VVL 113, VVL 114, VVL 122, and VVL 123) during all 16 sampling dates covering the dormant and active growth periods. The only exception was accession VVL-122, which was not tested on March 1, because the sample was spoiled under inadequate storage conditions. Cq values for GVG and 18S rRNA determined for different sampling dates and between different grapevine accessions, as well as their mean values and standard deviations, are provided in Supplementary Table S2.

**Figure 3:**
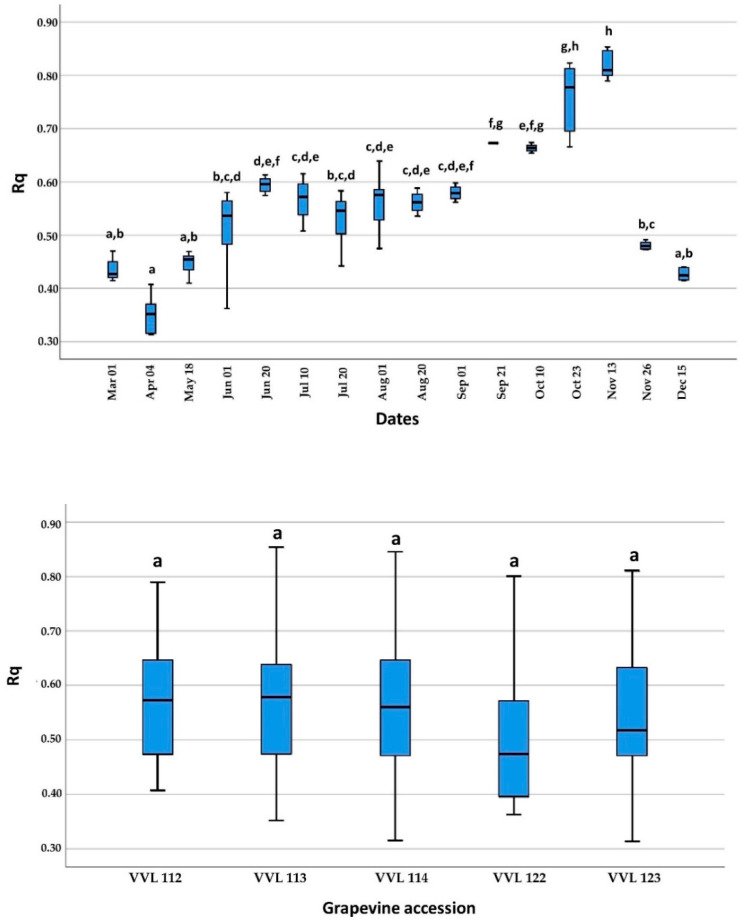
Display of relative quantification (Rq) and standard deviations of GVG in respect to the 18S rRNA used as internal control. A comparison was done between samples collected on 16 sampling dates during the 2019 growing season (**up**) and five different grapevine accessions (**down**). Different letters (a–h) represent a significant difference among collecting dates and grapevine accessions based on the Tukey HSD test (*p* < 0.05).

The Rq values obtained on April 4, the first sampling date at the beginning of active growing, were significantly lower compared to those obtained between 1 June and 26 November, but without significant differences when compared to other sampling dates (1 March, 18 May and 15 December). In addition, Rq values near the end of the active growing season (21 September and 23 October) were significantly higher than those obtained between 1 March and 20 August and 26 November and 15 December, except for the values on 1 September and 10 October. The highest Rq values were recorded on 13 November, the end of the active growing season, and were significantly higher than all sampling dates except 23 October. Finally, during dormancy, mid-November to March, there were no significant differences between the mean Rq values. A comparison of mean Rq values among the different grapevine accessions showed no significant differences (Figure 3).

### 2.3. Field Survey

In four collection plantations in Zagreb (continental Croatia) with 878 vines tested, 30 vines of autochthonous cultivars showed to be GVG-positive (3.4%), of which 22 were found in the Grapevine Virus Collection and 8 in the second National Collection in “Jazbina”. The presence of virus was not confirmed in the first National Collection in “Jazbina”, along with the Grapevine and Rootstock Collection. In the Split Collection, the only one located in the coastal region, out of 105 analyzed samples, 5 vines representing five different autochthonous cultivars were found to be infected (4.8%), giving the overall infection rate in the collection plantations of 3.6% (35/983).

Screening performed in 93 commercial vineyards, 16 located in the continental region (441 vines/samples) and 77 in the coastal region (2903 vines/samples), revealed no presence of GVG in the continental area, while in the coastal region, virus was confirmed in 421 (~14.5%) grapevines from 22 locations (28.6%). Considering the individual locations, the highest incidence of GVG was found in the locations Furnaže and Marceline (Split-Dalmatia County—Kaštela region) and Pag 4 (Zadar County), where a total of 50, 54, and 30 grapevines of cvs. Mladenka, Vlaška and Paška maraština were GVG-positive, respectively. Besides the mentioned autochthonous cultivars, infection incidence above 50% was also found in cultivars Cipar, Ljutun and Muškatel. The lowest infection rate was found in the location Zemunik Donji (Zadar County), with 2% infections in the autochthonous cv. Plavina, although the general infection rate of cv. Plavina was 32.5%. Of the 506 analyzed vines of the cv. Plavac mali, the most popular autochthonous grape variety in Croatia, only one (0.2%) was positive for GVG. Concerning the overall number of 4327 grapevines tested in this survey, GVG infection was detected in 456 samples, representing an overall infection rate of 10.5%. Interestingly, infection was not confirmed in imported cultivars and rootstocks included in the study. A detailed overview of the sampling vineyards together with GVG-positive vines and corresponding locations can be found in Supplementary Table S3.

**Figure 4:**
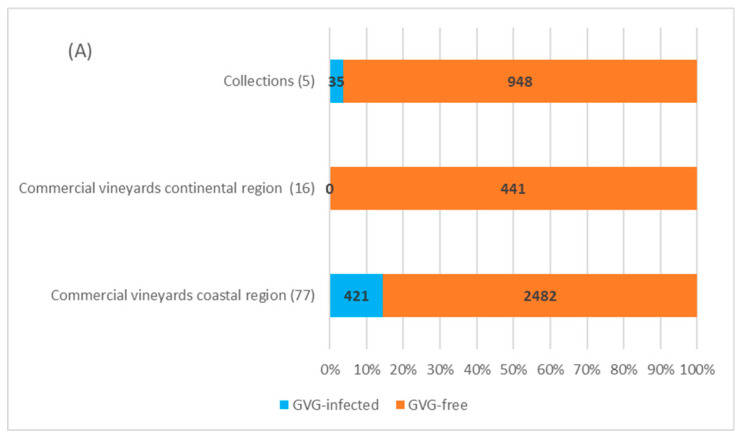

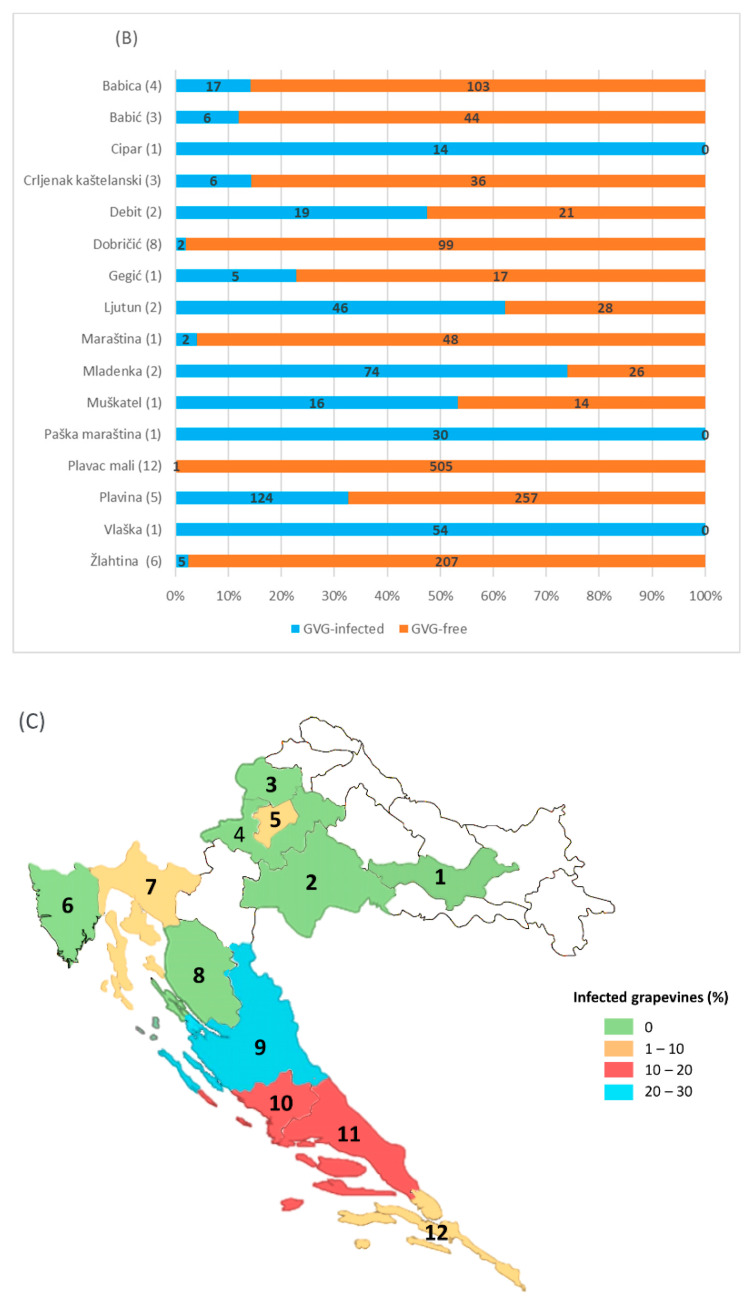
GVG infection determined in grapevine collections and commercial vineyards (**A**), in different grapevine varieties from commercial vineyards (**B**), and in different counties (**C**). The numbers in parenthesis indicate the number of sites/vineyards, while the numbers in the rows correspond to the number of samples/vines analyzed. Identifiers for the counties: 1—Požega-Slavonia; 2—Sisak-Moslavina; 3—Krapina-Zagorje, 4—Zagreb County; 5—City of Zagreb; 6—Istria; 7—Primorje-Gorski Kotar; 8—Lika-Senj; 9—Zadar; 10—Šibenik-Knin; 11—Split-Dalmatia; 12—Dubrovnik-Neretva. Unmarked counties (white) were not included in the survey.

Considering the regional distribution, GVG presence was confirmed in 6 of the 12 counties included in the survey. The highest infection rate was found in Zadar County—210/727 (28.9%), followed by Šibenik-Knin County (15%), Split-Dalmatia County (13%), Dubrovnik-Neretva County (4%), the City of Zagreb (3.3%) and Primorje-Gorski Kotar County (2.4%). The presence of the virus was not confirmed in other counties surveyed (Požega-Slavonia, Sisak-Moslavina, Krapina-Zagorje, Zagreb, Istria and Lika-Senj, Figure 4).

### 2.4. Phylogenetic Analysis Using Coat Protein Gene Sequence

Aiming to investigate the genetic variability of GVG based on the CP region, conventional one-step RT-PCR assay with corresponding primers was generated. Thus, in addition to the two previously described forward primers used in real-time RT-PCR (Table 1), a single reverse primer was designed: 5′-ACCTCCACAGGTCCTTCGG-3′ (Figure 1). Thirty-five GVG isolates, initially determined by real-time RT-PCR, were selected for amplification by conventional one-step RT-PCR with the mixture of the abovementioned three primers. Consequently, all isolates yielded amplicons of 606 nts, which were sequenced bidirectionally. The sequences of 564 nts (RT-PCR products without primers) were compared with each other, but also with already known GVG isolates from the USA, Croatia, and New Zealand present in GenBank, and the here-characterized two GVG isolates by HTS (VVL-150 and VM-160). A comparison of the sequences revealed 449 conserved, 115 variable, and 100 parsimony-informative sites (Supplementary Table S4), whereas the amino acid sequences consisted of 187 amino acids with 178 conserved, 9 variable, and 8 parsimony-informative sites (Supplementary Table S5). The highest differences at the nucleotide level were found in the Croatian isolates, where identities ranged from 89% to 100%, while at the amino acid level, identities ranged from 96.8% to 100%. The least differences were found between the New Zealand isolates, with identities at the nucleotide and amino acid levels ranging from 99.1 to 99.6%, and from 99.5 to 100%, respectively. Overall, nucleotide and amino acid identities between Croatian and New Zealand isolates ranged from 89.4 to 91.1% and from 97.3 to 100%, respectively.

**Figure 5:**
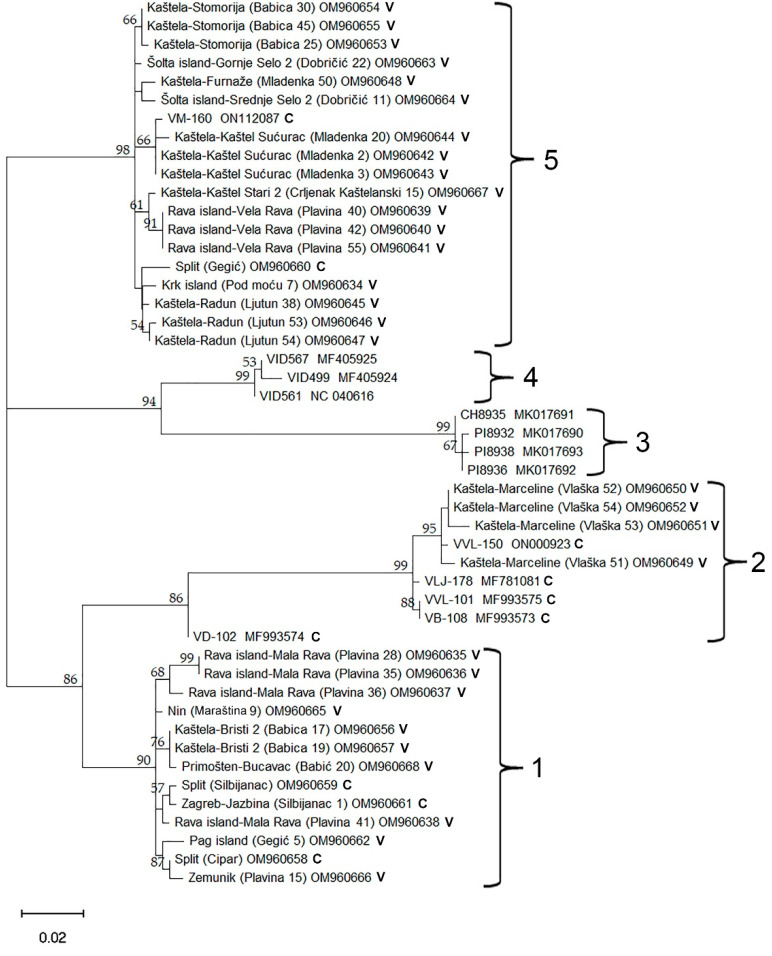
Maximum Likelihood (ML) tree showing phylogenetic relationships based on a 564 nts long sequence of the coat protein (CP) coding region of 37 newly discovered Croatian GVG isolates and 11 isolates from GenBank (VID561 (NC_040616), VID567 (MF405925) and VID499 (MF405924) from New Zealand; VLJ-178 (MF781081), VVL-101 (MF993575), VD-102 (MF993574) and VB-108 (MF993573) from Croatia; PI8936 (MK017692), PI8932 (MK017690), PI8938 (MK017693), and CH8935 (MK017691) from the USA). The ML tree was constructed using MEGA 11 with the Tamura 3-parameter + Gamma distribution (T3 + G) model of nucleotide substitution. The isolates were named according to region, vineyard location, cultivar, number of plants in the vineyard and corresponding GenBank accession number; C—collection plantation, V—commercial vineyard. Only results showing branch support above 50% are shown.

Phylogenetic analyses of the 37 newly sequenced Croatian GVG isolates, together with previously known isolates available in GenBank, reveal five distinct groups. Group 3 consists entirely of previously known isolates originating from the USA, while Group 4 consists of isolates from New Zealand. Group 2 included four previously known isolates from Croatia: VLJ-178, VVL-101, VD-102, and VB-108, and a newly discovered isolate via HTS (VVL-150), as well as four newly discovered isolates originating from the Kaštela-Marceline site. Groups 1 and 5 represent a previously unknown spectrum of GVG genetic diversity, which was revealed in this study (Figure 5). Nucleotide diversity (π) for the GVG species was estimated as 0.071. This is the probability that two randomly chosen isolates differ at any specific nucleotide. To state this differently, the GVG isolates analyzed were on average 92.9% identical at the nucleotide level. Nucleotide diversity for Croatian isolates alone was 0.061 compared to 0.052 for the international samples. Amongst the Croatian isolates, we observed similar levels of nucleotide diversity within the collection samples (π = 0.057) and the commercial vineyards (π = 0.068). The phylogenetic tree in Figure 5 suggests a high level of genetic structure within the GVG species. Using the five labeled clades as subpopulations that geographically separate GVG isolates, we estimated an Fst value of 0.87. We observe substantially more nucleotide diversity between clades (0.092) compared to within (0.012). When comparing Croatian isolates to those of New Zealand and the USA, we estimated an Fst value of 0.40, with more nucleotide diversity between groups (0.10) compared to within (0.061). Finally, we observed numerous instances of four gametes at pairs of bi-alleleic variant sites across the multiple alignment. While we cannot rule out the role of recurrent mutation and sequencing errors, this observation can most likely be explained by recombination.

## 3. Discussion

HTS has made great progress in the discovery of viruses in many plant species, including grapevine [22,23], where 10 new grapevine-infecting vitiviruses have been discovered in the last 11 years [5,6,7,8,9,10,11,12,13]. However, the presence of individual viruses, usually detected in one or a few samples, provides limited information about their genetic variability or their distribution and occurrence in a wider area. For these reasons, one of the main objectives of this work was to generate methods suitable for cost-effective large-scale testing, in this case a real-time RT-PCR assay. In validation and sensitivity analyses of this real-time RT-PCR assay, the amplification curve was obtained down to a dilution of 1:100,000. Lastly, although not presented here, real-time RT-PCR was found to be 1000-fold more sensitive than the conventional one-step RT-PCR used in this study. However, both assays were able to detect GVG in infected plant material involving different grapevine cultivars.

As demonstrated on five grapevine accessions, GVG can be detected by real-time RT-PCR throughout the growing season and the dormancy period using available plant tissue (petioles from basal leaves during vegetation and cortical scrapings during dormancy). In contrast, false-negative results for grapevine leafroll-associated virus 3 (GLRaV-3) [24,25] and grapevine red blotch virus (GRBV) [26] were observed in studies using the real-time PCR method based on petioles collected in early spring (April and May). In the aforementioned studies, this phenomenon was attributed to the low virus titer during this period, which was also confirmed in our study for GVG, with mean Rq values being lower at the beginning of vegetation, especially on April 4, using the shoots. There were no significant differences in Rq values between June 1 and September 1, but after this period (between 21 September and 13 November), Rq values began to increase (Figure 3). This can be attributed to the increase in Cq values of 18S rRNA due to leaf senescence. Interestingly, this leaf senescence was not accompanied by an increase in virus Cq values, considering that Cq levels are “negatively” correlated with virus titer (Supplementary Table S2). The same phenomenon was observed for GLRaV-1 and GLRaV-3, with high titers in leaves collected in October [27]. According to the results obtained for grapevine accession VVL 122, with the highest Cq values in 9 out of 16 sampling dates compared to the other four grapevine accessions, this opened the possibility of significant Cq value variations in individual plants during dormancy and the growing season (Supplementary Table S2).

A field survey done in commercial vineyards confirmed the presence of GVG in ~14.5% vines. Compared to similar studies done for other vitiviruses in Croatia, the occurrence of GVG is less than the determined infection with GVA (61.4%), but significantly higher compared to GVB (3.1%) [28]. Infection with GVG in grapevine collections (7.1% in the National Collection “Jazbina” and 4.8% in the Split Collection) could have an influence on further spread of GVG since, in the case of some endangered autochthonous cultivars, those plants are usually used as mother plants in revitalization programs. Furthermore, in analyzing the autochthonous and introduced cultivars, the presence of GVG was confirmed only in cultivars considered as autochthonous, although 17.4% of the analyzed samples belonged to introduced cultivars or rootstocks. Comparing two viticultural regions, GVG was not found in commercial vineyards in the continental region, but just in the two collection plantations and only on cultivars typical for the coastal wine-growing region. The fact that GVG was detected in 22 out of 77 commercial vineyards in the coastal region (28.6%) shows that the occurrence of GVG in Croatia is not sporadic, especially since the virus was detected in a percentage between 10 and 20% in Šibenik-Knin and Split-Dalmatia Counties and between 20 and 30% in Zadar County (Figure 4). Such a high incidence of GVG is comparable with the incidence of the economically important GLRaV-1 in Croatia, with an identified infection rate of 16.1% in autochthonous grapevines from the coastal region and 17.2% in vineyards of the Istrian peninsula [29,30]. A surprisingly low infestation rate of just 0.2% (1 positive sample out of 506 tested) was found in the Croatian most popular red-berry cultivar Plavac mali, compared to some other cultivars of local importance such as Paška maraština (100%), Vlaška (100%), Mladenka (74%), especially since previous studies conducted on Plavac mali have shown a deteriorated sanitary status of this cultivar associated with high infection rates by economically important viruses such as GVA, GLRaV-1, -3 and grapevine fanleaf virus (GFLV) [28,29,31]. Finally, the absence or very low infection rate determined on some locations (Zemunik Donji 2%, Kaštel Stari 1 2.5%) is comparable with GVG prevalence found on grapevine samples from Californian vineyards (USA), revealing the virus presence in just 4 out of 2436 analyzed vines [20].

Phylogenetic analyses performed on the 37 newly discovered and characterized Croatian GVG isolates, and 11 previously known isolates from the GenBank, separate the isolates into five groups. While GenBank isolates from the USA and New Zealand were assigned each into distinct groups (3 and 4), isolates from Croatia were dispersed into three groups (1, 2 and 5). Interestingly, Group 2 comprised previously known isolates from Croatia, together with five newly discovered isolates (Vlaška 51 OM960649, Vlaška 52 OM960650, Vlaška 53 OM960651, Vlaška 54 OM960652 and VVL-150 ON000923), all isolates in the mentioned group originating from the Kaštela region. Other newly characterized GVG isolates were classified into two different groups (1 and 5), representing novel, not known, genetic diversity of GVG. Thus, Group 5 showed to be the most abundant in the number of isolates, while Group 1 includes the largest number of isolates originating from different sites/vineyards. Another particularity noticeable from the phylogenetic tree was the grouping of isolates collected from the same site/vineyard close to each other. Such grouping suggests possible common ancestry and/or on-site transmission by insect vectors. The possibility of insect transmission could be assumed, especially since some other grapevine-infecting vitiviruses (i.e., GVA, GVB, GVE and GVH) can be successfully transmitted by various mealybugs (*Hemiptera*: *Pseudococcidae*) and soft-scale insects (*Hemiptera*: *Coccidae*) [18,32]. Additionally, the nucleotide diversity of GVG (π = 0.071) was within the range of those reported for other vitiviruses, with estimated values of π = 0.17, 0.008, 0.090 for GVB, GVE and GVF, respectively [33]. The Fst value for the five GVG phylogenetic clades (0.87) indicates highly restricted gene flow between those groups. Similarly, Fst values of 0.33, 0.50, and 0.49 for the phylogenetic clades of GVB, GVE, and GVF were reported [33]. The Fst value for comparing Croatian GVG isolates to the rest of the world was 0.40, consistent with Croatian isolates being distinct from those observed internationally. Similar values (Fst = 0.32 and 0.27) were observed when comparing Iranian vitivirus isolates of GVE and GVF to international isolates [33]. In contrast, the Iranian GVB isolates were not distinct from the rest of the world (Fst = 0.02).

Ultimately, this study is evidence of the wide distribution of GVG in Croatian autochthonous grapevine cultivars originating and/or grown along the Croatian coastal grapevine growing region. This may be important since some vitiviruses associated with RW disease can cause significant losses in grapevine production due to their effects on grapevine physiology, growth, yield, propagation, and wine quality. In previous studies, vitiviruses were often found together with economically important viruses from the leafroll complex. Viruses from these two complexes are thought to have specific interactions with each other and with the same vectors, allowing better transmission by coinfection [34]. According to the current Croatian legislation for grapevine planting material, plants used for propagation must be free from arabis mosaic virus, GFLV, GLRaV-1 and GLRaV-3, while vitiviruses are not regulated. In contrast, in other countries with a long viticultural tradition, vitiviruses with known adverse effect are regulated in order to prevent or, at least, slow down their spread. While producers can obtain virus-free planting material of introduced cultivars from abroad, the planting material of autochthonous cultivars is mainly restricted to domestic production. Thus, for cultivars of local importance, the risk of spread is increased by the limited number of vines used for propagation, usually selected from commercial vineyards or grapevine collections. All this could contribute to the further spread of GVG in Croatia in the future.

With this study, we expanded the knowledge of GVG biodiversity and developed a new diagnostic tool based on the RT-PCR method that can be used in monitoring programs. Considering the confirmed GVG infection rate of 10.5% in Croatia and the proven negative impact of some other vitiviruses (especially GVA and GVB) on grapevine, we have created the necessary prerequisites for future research aimed at a better understanding of GVG ecology, the impact on grapevine production and, consequently, possible inclusion in certification schemes.

## 4. Materials and Methods

### 4.1. Real-Time RT-PCR Assay, HTS and Bioinformatic Analysis

For the construction of primers and probes, 11 sequences available from the GenBank (isolates VID561—NC_040616, VID567—MF405925, VID499—MF405924, VLJ-178—MF781081, VVL-101—MF993575, VD-102—MF993574, VB-108—MF993573, PI8938—MK017693, PI8936—MK017692, CH8935—MK017691, and PI8932—MK017690), along with two GVG-positive vines from the Grapevine Virus Collection in Zagreb determined during this study, were used. To obtain insight into full-genome sequence, those two GVG-infected grapevine accessions were subjected to total nucleic acids (TNA) extraction and HTS, according to the previously described protocol [35]. Thus, constructed cDNA libraries were sequenced on an Illumina NextSeq 500 platform at the University of California-Davis. After the demultiplication and adapter removal by bcl2fastq software (Illumina, San Diego, CA, USA), de novo assembly was done using SPADes [36]. Assembled contigs were annotated via BLASTn and BLASTx. Later, the obtained GVG sequences, along with 11 previously mentioned isolates from the GenBank, were used for primers and probe construction using the Primer 3 (https://primer3.org/webinterface.html, accessed on 5 August 2019) and Geneious 10.2.6 (https://www.geneious.com, accessed on 5 August 2019) programs.

The real-time RT-PCR assay, which included 18S rRNA oligos as an internal control [37], was prepared in a 20 µL reaction volume that consisted of: 0.4 µM of each primer, 0.15 µM of the probe, 5 µL of TaqMan™ Fast Virus 1-Step Master Mix (Applied Biosystems, Thermo Fischer Scientific, Waltham, MA, USA), 10.6 µL of ultrapure water, and 2 µL of RNA as a template. Reaction conditions were as follows: the initial activation step was 10 min at 95 °C, followed by 40 cycles at 94 °C for 15 s, and the elongation step at 60 °C for 1 min. Reactions were performed in the Applied Biosystems 7500 Real-Time PCR System (Thermo Fischer Scientific, Waltham, MA, USA).

Isolation of RNA used in the above-mentioned assay was done by the previously described glycine-EDTA-sodium (GES) method [38] using 0.1 g of leaf petioles crushed to a fine powder in mortar with a pestle and liquid nitrogen. Homogenized plant powder was transferred to 2 mL tubes with the addition of 1.8 mL of grinding buffer (0.015 M Na_2_CO_3_, 0.035 M NaHCO_3_, 0.0005 M PVP, 0.2% bovine serum albumin, 0.05% Tween 20, pH 9.6 with acetic acid). After centrifugation at 13,200× *g* for 10 min, the supernatant was transferred to a new 2 mL collection tube. In a 100 µL of GES buffer (0.1 M glycine, 50 mM NaCl, 1 mM EDTA, 0.5% Triton X, 1% β-mercaptoethanol, pH 9.0 with NaOH) prepared, 8 µL of extract were added and denaturized in Mastercycler (Eppendorf, Hamburg, Germany) at 95 °C for 10 min. Subsequently, the availability of nucleic acids was checked spectrophotometrically on NanoPhotometer P330 (Implen, Munich, Germany).

To validate the new real-time RT-PCR, a GVG-positive grapevine from the Grapevine Virus Collection was selected and subjected to RNA extraction. Sensitivity and validation comparison was performed with 10-fold serial dilutions, ranging from 1 to 100,000, in water and prepared in three replicates. The reactions consisted of 2 µL of each dilution in 20 µL of the final volume. Finally, the efficiency of the assay was evaluated using the results of a standard curve (Applied Biosystems 7500 software, ver. 2.3).

### 4.2. Detection during Dormancy and Growing Season

To obtain real-time RT-PCR detection capability during active growth and dormancy, five GVG-infected grapevine accessions of the cultivar Vlaška (VVL-112, VVL-113, VVL-114, VVL-122, and VVL-123) were selected from the Grapevine Virus Collection. Sampling and RNA isolation were performed at 16 different time points: at the beginning of vegetation from shoots (April), during vegetation from leaf petioles (mid-May to mid-November) and from cortical scrapings during dormancy (mid-November to March). Quantification cycle (Cq) values for GVG and 18S rRNA were measured and processed with the software IBM SPSS Statistics, ver. 25 [39]. In addition, the determined Cq values were used for relative quantification (Rq) using the following formula:

Rq = Cq(18S rRNA)/Cq(GVG)

The Kolmogorov–Smirnov (KS) and Shapiro–Wilk tests were used to evaluate normality [40,41]. One-way analysis of variance (ANOVA) was used to compare determined values, while differences between collection dates and grapevine accessions were evaluated by the post hoc Tukey test [42].

### 4.3. Field Survey

As part of the study on the incidence and distribution of GVG in Croatia, samples of autochthonous and introduced grapevine cultivars were collected in June/July 2020 and 2021, both from collection plantations and commercial vineyards along the Croatian continental and coastal grapevine growing regions (Supplementary Table S3). In the absence of data on GVG symptomatology in grapevines, samples were randomly selected. Three leaf petioles were taken from different parts of the canopy from each grapevine included in the survey, and RNA was extracted as previously described, followed by real-time RT-PCR detection. The sampling strategy and selection of cultivars was adjusted to their importance, especially those considered as autochthonous, and the significance of viticulture in the different regions. In the continental part, 441 samples from 16 commercial vineyards from four counties (Požega-Slavonia, Sisak-Moslavina, Krapina-Zagorje, and Zagreb), together with 878 samples from four collection plantations located in the city of Zagreb (Grapevine Virus Collection—196 vines, Grapevine and Rootstocks Collection—91 vines and two national collections of autochthonous Croatian cultivars in the experimental station “Jazbina”—together 591 vines), were included in the study. From the coastal grapevine growing area, 2903 samples from 77 commercial vineyards from seven counties (Istria, Primorje-Gorski Kotar, Lika-Senj, Zadar, Šibenik-Knin, Split-Dalmatia and Dubrovnik-Neretva) and 105 vines from a collection plantation in Split (Institute for Adriatic Crops and Karst Reclamation) were analyzed. In total, 4327 vines originating from 93 commercial vineyards and 5 collection plantations were analyzed. Most of the samples (3574; 82.6%) were from Croatian autochthonous cultivars, while 753 (17.4%) were introduced cultivars and rootstocks, including Graševina as cultivar with uncertain origin.

### 4.4. Phylogenetic Analysis Using Coat Protein Gene Sequence

To get insight into sequence homology of different GVG isolates, conventional one-step RT-PCR assay was used; thus, primers covering almost all the coat protein (CP) region (amplicon size 606 bps, 564 bps after primers removal) were constructed using the data from the same sequences as for the design of primers and the probe for real-time RT-PCR. The reaction was prepared in a 25 µL mixture using a One-step RT-PCR kit (Qiagen, Hilden, Germany), according to manufacturer’s recommendation, with the addition of 0.2 µL of isolated RNA as a template and 0.4 µM of each primer. Reactions were performed in a Mastercycler (Eppendorf, Hamburg, Germany) under the following conditions: reverse transcription at 50 °C for 30 min, initial activation step at 95 °C for 15 min, 35 cycles at 94 °C for 30 s, 55 °C for 30 s, 72 °C for 1 min, and a final elongation step at 72 °C for 10 min. 1X TBE buffer was used to prepare a 1.5% agarose gel with one drop of GelRed (CareDx AB, Stockholm, Sweden). Lastly, amplicons were verified by horizontal gel electrophoresis and visualized on the UV Transilluminator 2000 (Bio-Rad, Hercules, CA, USA). Thirty-five GVG-positive plants identified during the field survey and originating from 20 different vineyards/sites were selected for conventional one-step RT-PCR, followed by direct sequencing. Multiple grapevines (two or three) were sampled from nine sites (Mala Rava, Vela Rava, Kaštel Sućurac, Radun, Marceline, Stomorija, Bristi 2, the Grapevine Virus Collection in Zagreb and the Collection plantation in Split) to obtain information on GVG genetic diversity within the same sites, while one grapevine was sampled from 11 sites (Gornje selo 2, Srednje selo 2, Furnaže, Kaštel Stari 2, Pod moću, Nin, Bucavac, Jazbina 2, Pag island, Zemunik, Jazbina) to obtain information on variance among sites. Sanger sequencing was performed in both directions at Macrogen Europe (Amsterdam, The Netherlands). The sequences were processed in Bioedit 7.2. [43]. The 35 consensus sequences were phylogenetically compared, together with two here-sequenced isolates by HTS and 11 isolates from the GenBank, originating from New Zealand (3), Croatia (4) and the USA (4). MEGA11 software was used for both nucleotide and amino acid level comparisons using the p-distance method and for phylogenetic tree construction using the maximum likelihood (ML) method with 1000 bootstrap repeats and the nucleotide substitution model with the lowest BIC score [44]. Additionally, we characterized sequence variation by computing estimating population genetic parameters on the GVG multiple alignment (Supplementary Tables S4 and S5). Nucleotide diversity (π) for the virus species as well as subpopulations was estimated using the method of Nei and Gojobori [45]. To further quantify genetic structure, we estimated the fixation index (Fst) using the method of Hudson et al. [46]. All estimates were done on a complete and contiguous portion of the multiple alignment from positions 1 to 474.

## Figures and Tables

**Table 1:** Primers and probes designed for GVG detection by real-time RT-PCR.

Primer/Probe Name	Orientation	Target Gene	Primer Sequence (5′–3′)
GVG F1	Forward	Coat Protein (CP)	AAGGATGGGAGAGGAATCAAAGA
GVG F2	Forward		AAGGATGGGAGAGGAATCCAAG
GVG R1	Reverse		TTTAGAGCCGGGTGCTTCAC
GVG R2	Reverse		TAGAGCCGGATGCTTCACG
GVG R3	Reverse		TTTAGGGCCGGGTGCTTC
GVG P	TaqMan probe		NED-AGCAGTACTGACGCTGCT-MGB

## Data Availability

All sequencing data of Croatian GVG isolates obtained in this study were included in the manuscript and/or submitted to the GenBank database under the accession numbers: OM960634—OM960668, ON000923 and ON112087.

## Supplementary Materials

The following supporting information can be downloaded at https://www.mdpi.com/article/10.3390/plants11182341/s1, Table S1: Sequences of newly characterized GVG isolates determined by HTS. Table S2: Cq values obtained by real-time RT-PCR for GVG and 18S rRNA in different grapevine accessions at different sampling dates, including mean values, standard deviations, and standard errors. Table S3: Number of grapevine samples included in the survey by county, region, vineyard location, and cultivar in the continental and the coastal parts of Croatia. Tables S4 and S5: Multiple sequence alignment of the partial coat protein gene (564 nts/187 aa) from 37 GVG isolates characterized in this study and 11 isolates from the GenBank, originating from New Zealand, Croatia, and USA.
